# Delays before Diagnosis and Initiation of Treatment in Patients Presenting to Mental Health Services with Bipolar Disorder

**DOI:** 10.1371/journal.pone.0126530

**Published:** 2015-05-20

**Authors:** Rashmi Patel, Hitesh Shetty, Richard Jackson, Matthew Broadbent, Robert Stewart, Jane Boydell, Philip McGuire, Matthew Taylor

**Affiliations:** 1 King’s College London, Department of Psychosis Studies, Institute of Psychiatry, Psychology & Neuroscience, London, United Kingdom; 2 South London and Maudsley NHS Foundation Trust, Biomedical Research Centre Nucleus, London, United Kingdom; 3 King’s College London, Department of Psychological Medicine, Institute of Psychiatry, Psychology & Neuroscience, London, United Kingdom; University of South Florida, UNITED STATES

## Abstract

**Background:**

Bipolar disorder is a significant cause of morbidity and mortality. Although existing treatments are effective, there is often a substantial delay before diagnosis and treatment initiation. We sought to investigate factors associated with the delay before diagnosis of bipolar disorder and the onset of treatment in secondary mental healthcare.

**Method:**

Retrospective cohort study using anonymised electronic mental health record data from the South London and Maudsley NHS Foundation Trust (SLaM) Biomedical Research Centre (BRC) Case Register on 1364 adults diagnosed with bipolar disorder between 2007 and 2012. The following predictor variables were analysed in a multivariable Cox regression analysis: age, gender, ethnicity, compulsory admission to hospital under the UK Mental Health Act, marital status and other diagnoses prior to bipolar disorder. The outcomes were time to recorded diagnosis from first presentation to specialist mental health services (the diagnostic delay), and time to the start of appropriate therapy (treatment delay).

**Results:**

The median diagnostic delay was 62 days (interquartile range: 17–243) and median treatment delay was 31 days (4–122). Compulsory hospital admission was associated with a significant reduction in both diagnostic delay (hazard ratio 2.58, 95% CI 2.18–3.06) and treatment delay (4.40, 3.63–5.62). Prior diagnoses of other psychiatric disorders were associated with increased diagnostic delay, particularly alcohol (0.48, 0.33–0.41) and substance misuse disorders (0.44, 0.31–0.61). Prior diagnosis of schizophrenia and psychotic depression were associated with reduced treatment delay.

**Conclusions:**

Some individuals experience a significant delay in diagnosis and treatment of bipolar disorder after initiation of specialist mental healthcare, particularly those who have prior diagnoses of alcohol and substance misuse disorders. These findings highlight a need for further study on strategies to better identify underlying symptoms and offer appropriate treatment sooner in order to facilitate improved clinical outcomes, such as developing specialist early intervention services to identify and treat people with bipolar disorder.

## Introduction

Bipolar disorder is a major global health problem, associated with increased mortality[1,2], substantial disability[3], and major societal economic impact[4]. However people with bipolar disorder often experience delays of several years before receiving a diagnosis and appropriate treatment. Retrospective studies from USA and Australia have found that people with bipolar disorder report delays of more than five years, often more than ten years, between the onset of illness and diagnosis[5,6]. Moreover, studies from specialist centres typically find there to have also been substantial delays of many years before initiation of appropriate medication[7–10].

Delayed diagnosis and treatment of bipolar disorder are associated with poorer long term outcomes[8,10,11], and repeated episodes of bipolar disorder are associated with increased vulnerability to relapse[12] and deteriorations in cognitive function[13]. Furthermore, the failure to correctly recognise underlying bipolar disorder may lead to an individual receiving inappropriate treatment such as antidepressant monotherapy which may be associated with increased risk of developing mania[14,15].

One of the barriers to the prompt recognition and management of this group may be a failure to recognise symptoms indicative of underlying bipolar disorder[16], and the attribution of these to another mental illness[17] or comorbid substance misuse[18]. Furthermore, the nature of psychopathology experienced by an individual with bipolar disorder can vary significantly over the course of illness[19]. In the early stages, the pattern of symptoms may not be well characterised by criterion-based ICD/DSM diagnostic classification potentially leading to a delay in receiving appropriate treatment[20].

An improved understanding of factors associated with delay to diagnosis and delay to treatment would inform the development of strategies to reduce them. In the present study, we analysed clinical data from individuals with bipolar disorder in a large geographically-defined community in order to estimate the delay to diagnosis and initiation of appropriate treatment after presentation to mental health services. Our first hypothesis was that even when they are assessed by mental health teams, there are still significant delays before patients with bipolar disorder receive a diagnosis and appropriate treatment. We also investigated the factors associated with delays to diagnosis and treatment, and tested the hypothesis that these delays would be longer in patients who had previously been diagnosed with other mental illnesses or comorbid alcohol and substance misuse.

## Methods

### Participants

Data were obtained from the South London and Maudsley NHS Foundation Trust (SLaM) Biomedical Research Centre Case Register. Under the National Health Service (NHS) system in the UK, there is universal state provision of healthcare with Mental Health Trusts providing specialist mental healthcare to people living in defined geographic catchment areas[21]. In order to obtain specialist mental healthcare, patients typically consult their local general practitioner (a provider of primary healthcare) who may then initiate a referral to the respective specialist mental healthcare service. Alternatively, patients may be referred to specialist mental healthcare services after presenting to emergency departments and some services accept direct patient referrals. Initial assessments may be performed by a trainee or consultant psychiatrist or other mental healthcare professional[21]. The South London and Maudsley NHS Foundation Trust (SLaM) is one of Europe’s largest provider of secondary mental healthcare, serving four boroughs in southeast London (urban and suburban areas) with a geographic catchment of approximately 1.2 million residents and provision of all aspects of secondary mental healthcare to all age groups including inpatient, community, general hospital liaison and forensic services. Fully electronic clinical records have been implemented in all SLaM services since 2007, and an electronic case register (the SLaM BRC Case Register) has been developed by the SLaM BRC for Mental Health rendering anonymised electronic clinic records data available for research on over 250,000 people receiving care from SLaM[22], with a robust patient-led governance framework. The SLaM Case Register has been approved as an anonymised data resource for secondary analyses by Oxfordshire Research Ethics Committee C (08/H0606/71+5) and governance is provided for all projects by a patient-led oversight committee in the Biomedical Research Centre, South London and Maudsley NHS Trust[23].

Using the SLaM BRC Case Register, a cohort of 1364 individuals meeting the following criteria were identified:

- First presentation to SLaM between 1^st^ January 2007 and 31^st^ December 2012 to an inpatient or community mental health service.
- Age between 16 and 65 years at first presentation.
- Subsequent diagnosis of mania or bipolar affective disorder before 31^st^ December 2013 defined according to ICD-10 diagnostic categories F30.x and F31.x.

Of these, 344 (25.2%) presented initially to inpatient services and the remainder presented to community services.

In order to ensure that participants included in the analysis had a stable diagnosis of bipolar disorder, only participants whose mania or bipolar disorder diagnosis was confirmed at least once within one year of initial diagnosis were included. The time period of 2007 to 2012 was chosen as 2007 was the first full year in which electronic health records were implemented in all SLaM services (thereby ensuring a representative clinical sample of individuals receiving specialist mental healthcare) and to ensure that all individuals in the study had at least one year of follow-up data available.

### Source of clinical data

Data were extracted from the BRC Case Register using the Clinical Record Interactive Search (CRIS) application[22]. The CRIS application is a bespoke software package which permits focussed searching of anonymised electronic health records in the SLaM BRC Case Register and has contributed to a wide range of epidemiological studies of mental disorder outcomes[1,24,25]. The records include structured fields for demographic information and clinical questionnaires as well as unstructured free text to record history, mental state examination, diagnostic formulation and management plan. The clinical data are those recorded contemporaneously by mental health professionals during routine clinical care and included correspondence between healthcare professionals such as clinic letters and discharge summaries. In order to maximise ascertainment of diagnosis and medication, data on these variables were obtained from unstructured free text clinical entries (as well as structured text fields) using natural language processing (NLP)[26]. All other variables were ascertained from structured fields.

### Predictor variables

The CRIS application was used to extract the following predictor variables from the dataset: whether admitted to hospital compulsorily under the UK Mental Health Act (MHA) within 2 weeks of first presentation to SLaM, and diagnoses of schizophrenia and related disorders (ICD-10 F2x), psychotic depression (ICD-10 F32.3/F33.3), unipolar depression without psychotic symptoms (ICD-10 F32/33 excluding F3x.3), anxiety disorder (ICD-10 F4x), personality disorder (ICD-10 F60/F61), alcohol misuse/dependence (ICD-10 F10.x) or illicit drug misuse/dependence (ICD-10 F11-19.x) recorded prior to the date of first bipolar disorder diagnosis.

The following variables were extracted as covariates for multivariable analyses: age, gender, ethnicity and marital status. All covariate data obtained were those closest to the time of first referral to SLaM.

### Outcome variables

The primary outcome variable was time to diagnosis of bipolar disorder (in days) measured from the date of first presentation to SLaM. We considered this time to represent the delay to diagnosis of bipolar disorder while receiving specialist mental healthcare. The secondary outcome variable was time to first prescription of appropriate treatment (in days) measured from the date of first presentation to SLaM. We considered this time to represent the delay to initiating treatment. Following previous work in this area[9] we defined appropriate pharmacological treatment with reference to the British Association of Psychopharmacology guidelines[27] as initiation of any of second generation antipsychotic, lithium, valproate, carbamazepine and lamotrigine[9].

### Statistical analysis

STATA (version 12) software was used[28]. Descriptive statistics for predictor and outcome variables were obtained as means and standard deviations for continuous variables (diagnostic delay and treatment delay) and as frequencies and percentages for all other variables. Associations between predictor/covariate variables and diagnostic delay/treatment delay were investigated using Kaplan-Meier survival analysis and multivariable Cox regression. Proportionality of hazards was tested on Schoenfeld residuals of diagnostic delay and treatment delay using the phtest command in STATA[28]. The hazard ratios in Cox regression analyses represent the probability of bipolar disorder diagnosis or initiation of treatment occurring during the period of follow-up. Therefore, a hazard ratio greater than 1.0 indicates an association of a predictor variable with reduced time to diagnosis or treatment compared to the reference category. Reference categories for Cox regression analysis were defined as those of greatest prevalence. In order to adjust for comorbidity between prior diagnoses, all prior diagnoses were entered as individual binary variables in the Cox regression analysis rather than analysing these as a single categorical variable. Bonferroni correction of confidence intervals and p values was performed to mitigate the possibility of a type 1 statistical error for categorical variables with more than one category. By virtue of the study inclusion criteria, all participants were diagnosed with bipolar disorder and so no censoring was required in the survival analysis of diagnostic delay. A further analysis of diagnostic delay using multiple linear regression was also performed as a sensitivity analysis. For analysis of treatment delay, the outcome of starting appropriate treatment was censored at 31^st^ December 2013. To investigate the impact of missing covariate data, the main analyses included missing data as a separate category and further sensitivity analyses were performed including only participants with full covariate data.

## Results

### Diagnostic delay

**Fig 1:**
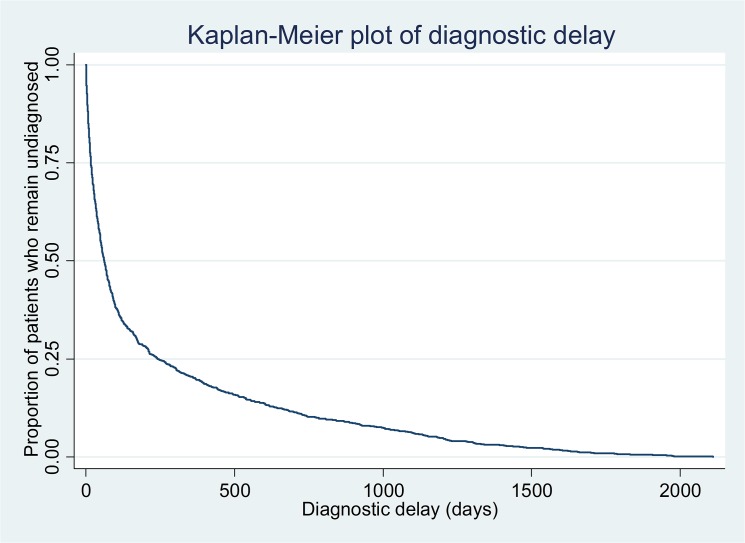
Kaplan-Meier plot of diagnostic delay.

The median delay to diagnosis of bipolar disorder was 62 days (IQR 17–243). Kaplan-Meier analysis (Fig 1) illustrates the distribution of diagnostic delay over time. Table 1 shows the breakdown of diagnostic delay according to demographic characteristics and diagnoses recorded prior to bipolar disorder. There were no significant differences in diagnostic delay depending on age, gender, ethnicity or marital status. Prior diagnoses of other psychiatric disorders were associated with increased diagnostic delay compared to people without these prior diagnoses. In particular, prior diagnoses of alcohol or substance misuse disorders were associated with substantially longer median delays to diagnosis. Compulsory admission under the UK Mental Health Act was associated with decreased diagnostic delay. These findings were corroborated on multivariable Cox Regression analysis. Multiple linear regression yielded comparable results (Table 2). A test of proportionality of hazards (Table 3) revealed a skewed distribution of diagnostic delay for compulsory admission, and prior diagnoses of schizophrenia and related disorders, unipolar depression without psychotic symptoms, anxiety disorders and illicit drug misuse or dependence. Although people with no recorded marital status were found to have shorter diagnostic delay, a sensitivity analysis including only those with full covariate data (Table 4) did not result in meaningful changes to the main findings.

**Table 1:** Factors associated with bipolar disorder diagnostic delay (n = 1364).

Factor	Group	Number in sample	Percentage	Median diagnostic delay in days (IQR)	*Adjusted hazard ratio	95% confidence interval, p value	Bonferroni corrected 95% confidence interval, p value
Age (years)	16–25	354	26.0%	75 (21–306)	0.93	0.80–1.08, p = 0.33	0.77–1.12, p = 1.0
	26–35	442	32.4%	58 (15–201)	Reference		
	36–45	314	23.0%	64 (21–262)	0.93	0.80–1.08, p = 0.36	0.77–1.13, p = 1.0
	46–55	158	11.6%	49 (12–164)	1.00	0.83–1.20, p = 0.99	0.79–1.27, p = 1.0
	56–65	96	7.0%	48 (13–360)	0.95	0.75–1.19, p = 0.64	0.71–1.27, p = 1.0
Gender	Female	796	58.4%	62 (16–240)	Reference		
	Male	644	47.2%	63 (21–256)	1.01	0.90–1.13, p = 0.88	0.90–1.13, p = 0.88
Ethnicity	White	837	61.4%	66 (18–262)	Reference		
	Asian	71	5.2%	63 (14–300)	0.75	0.58–0.99, p = 0.03	0.54–1.05, p = 0.13
	Black	239	17.5%	54 (12–301)	1.02	0.87–1.19, p = 0.84	0.83–1.25, p = 1.0
	Other	170	12.5%	56 (22–169)	1.07	0.90–1.26, p = 0.46	0.86–1.32, p = 1.0
	Ethnicity not recorded	47	3.4%	54 (19–148)	0.83	0.61–1.12, p = 0.22	0.56–1.22, p = 0.87
Marital status	Married/Cohabiting	318	23.3%	49 (14–211)	1.10	0.96–1.26, p = 0.19	0.92–1.31, p = 0.76
	Divorced/Separated	121	8.9%	79 (22–415)	0.94	0.76–1.15, p = 0.53	0.72–1.22, p = 1.0
	Single	824	60.4%	67 (19–270)	Reference		
	Widowed	12	0.9%	49 (12–140)	1.08	0.60–1.96, p = 0.79	0.51–2.30, p = 1.0
	Marital status not recorded	89	6.5%	35 (9–101)	1.45	1.16–1.83, p = 0.001	1.09–1.95, p = 0.005
UK Mental Health Act	Compulsory admission within 2 weeks of first presentation	232	17.0%	18 (6–70)	2.58	2.18–3.06, p<0.001	2.18–3.06, p<0.001
Prior Diagnoses	Schizophrenia or related disorders	215	15.8%	212 (48–670)	0.41	0.34–0.48, p<0.001	0.34–0.48, p<0.001
	Psychotic depression	37	2.7%	370 (93–929)	0.71	0.50–1.00, p = 0.05	0.50–1.00, p = 0.05
	Unipolar depression without psychotic symptoms	138	10.1%	409 (123–1051)	0.41	0.34–0.49, p<0.001	0.34–0.49, p<0.001
	Anxiety disorder	82	6.0%	442 (158–901)	0.52	0.41–0.65, p<0.001	0.41–0.65, p<0.001
	Personality disorder	54	4.0%	504 (169–1044)	0.59	0.45–0.79, p<0.001	0.45–0.79, p<0.001
	Alcohol misuse or dependence	27	2.0%	705 (356–1341)	0.48	0.33–0.71, p<0.001	0.33–0.71, p<0.001
	Illicit drug misuse or dependence	36	2.6%	742 (320–1142)	0.44	0.31–0.61, p<0.001	0.31–0.61, p<0.001

*Model adjusted for all factors listed in this table

**Table 2:** Factors associated with bipolar disorder diagnostic delay (n = 1364) analysed using multiple linear regression.

Factor	Group	Number in sample	Percentage	Median diagnostic delay in days (IQR)	*Adjusted B coefficient (days)	95% confidence interval, p value	Bonferroni corrected 95% confidence interval, p value
Age (years)	16–25	354	26.0%	75 (21–306)	6.4	-41.1, 53.9, p = 0.79	-54.1, 66.9, p = 1.0
	26–35	442	32.4%	58 (15–201)	Reference		
	36–45	314	23.0%	64 (21–262)	2.4	-46.5, 51.2, p = 0.92	-59.9, 64.6, p = 1.0
	46–55	158	11.6%	49 (12–164)	-24.3	-85.7, 37.1, p = 0.44	-102.6, 54.0, p = 1.0
	56–65	96	7.0%	48 (13–360)	28.2	-47.2, 103.6, p = 0.46	-67.9, 124.4, p = 1.0
Gender	Female	796	58.4%	62 (16–240)	Reference		
	Male	644	47.2%	63 (21–256)	-9.4	-45.3, 26.5, p = 0.61	-45.3, 26.5, p = 0.61
Ethnicity	White	837	61.4%	66 (18–262)	Reference		
	Asian	71	5.2%	63 (14–300)	51.4	-29.8, 132.7, p = 0.21	-52.1, 155.0, p = 0.86
	Black	239	17.5%	54 (12–301)	23.4	-26.8, 73.6, p = 0.36	-40.6, 87.5, p = 1.0
	Other	170	12.5%	56 (22–169)	-6.3	-60.9, 48.4, p = 0.82	-75.9, 63.4, p = 1.0
	Ethnicity not recorded	47	3.4%	54 (19–148)	49.1	-49.8, 147.9, p = 0.33	-77.0, 175.1, p = 1.0
Marital status	Married/Cohabiting	318	23.3%	49 (14–211)	-7.9	-53.6, 37.8, p = 0.73	-66.2, 50.3, p = 1.0
	Divorced/Separated	121	8.9%	79 (22–415)	23.3	-43.4, 90.0, p = 0.49	-61.7, 108.3, p = 1.0
	Single	824	60.4%	67 (19–270)	Reference		
	Widowed	12	0.9%	49 (12–140)	-1.4	-193.7, 191.0, p = 0.99	-246.6, 243.9, p = 1.0
	Marital status not recorded	89	6.5%	35 (9–101)	-80.4	-154.8, -6.1, p = 0.03	-175.2, 14.4, p = 0.14
UK Mental Health Act	Compulsory admission within 2 weeks of first presentation	232	17.0%	18 (6–70)	-144.3	-194.4, -94.2, p<0.001	-194.4, -94.2, p<0.001
Prior Diagnoses	Schizophrenia or related disorders	215	15.8%	212 (48–670)	214.8	163.6, 266.0, p<0.001	163.6, 266.0, p<0.001
	Psychotic depression	37	2.7%	370 (93–929)	129.7	17.4, 242.1, p = 0.02	17.4, 242.1, p = 0.02
	Unipolar depression without psychotic symptoms	138	10.1%	409 (123–1051)	352.6	292.1, 413.0, p<0.001	292.1, 413.0, p<0.001
	Anxiety disorder	82	6.0%	442 (158–901)	235.6	160.1, 311.2, p<0.001	160.1, 311.2, p<0.001
	Personality disorder	54	4.0%	504 (169–1044)	242.6	148.3, 336.9, p<0.001	148.3, 336.9, p<0.001
	Alcohol misuse or dependence	27	2.0%	705 (356–1341)	461.8	334.5, 589.1, p<0.001	334.5, 589.1, p<0.001
	Illicit drug misuse or dependence	36	2.6%	742 (320–1142)	363.0	249.9, 476.1, p<0.001	249.9, 476.1, p<0.001

*Model adjusted for all factors listed in this table

**Table 3:** Test of proportionality of hazards on Schoenfeld residuals for diagnostic delay.

Factor	Group	Rho	χ^2^	P value
Age (years)	16–25	0.04	2.6	0.11
	26–35	Reference		
	36–45	0.04	2.4	0.12
	46–55	0.01	0.1	0.77
	56–65	-0.03	1.5	0.22
Gender	Female	Reference		
	Male	0.03	0.9	0.35
Ethnicity	White	Reference		
	Asian	-0.04	2.6	0.11
	Black	0.01	0.05	0.83
	Other	0.06	5.8	0.02
	Ethnicity not recorded	-0.02	0.5	0.48
Marital status	Married/Cohabiting	0.01	0.3	0.59
	Divorced/Separated	0.00	0.0	0.89
	Single	Reference		
	Widowed	0.01	0.1	0.71
	Marital status not recorded	0.03	1.1	0.29
UK Mental Health Act	Compulsory admission within 2 weeks of first presentation	-0.14	31.4	<0.001
Prior Diagnoses	Schizophrenia or related disorders	0.14	28.6	<0.001
	Psychotic depression	0.05	2.9	0.09
	Unipolar depression without psychotic symptoms	0.12	17.9	<0.001
	Anxiety disorder	0.12	20.9	<0.001
	Personality disorder	0.06	4.0	0.05
	Alcohol misuse or dependence	0.04	1.6	0.20
	Illicit drug misuse or dependence	0.08	8.7	0.003

**Table 4:** Factors associated with bipolar disorder diagnostic delay only including participants with complete covariate data (n = 1244).

Factor	Group	Number in sample	Percentage	Median diagnostic delay in days (IQR)	*Adjusted hazard ratio	95% confidence interval, p value	Bonferroni corrected 95% confidence interval, p value
Age (years)	16–25	324	23.8%	70 (20–308)	0.99	0.85–1.15, p = 0.89	0.81–1.20, p = 1.0
	26–35	404	29.6%	62 (16–214)	Reference		
	36–45	277	20.3%	70 (23–327)	0.97	0.83–1.14, p = 0.73	0.79–1.19, p = 1.0
	46–55	150	11.0%	51 (14–166)	1.02	0.84–1.23, p = 0.87	0.79–1.30, p = 1.0
	56–65	89	6.5%	49 (13–390)	0.95	0.75–1.20, p = 0.66	0.70–1.28, p = 1.0
Gender	Female	734	53.8%	65 (16–267)	Reference		
	Male	510	37.4%	64 (21–297)	1.01	0.90–1.13, p = 0.87	0.90–1.13, p = 0.87
Ethnicity	White	785	57.6%	68 (19–282)	Reference		
	Asian	66	4.8%	70 (17–308)	0.2	0.55–0.94, p = 0.02	0.51–1.00, p = 0.05
	Black	232	17.0%	55 (13–301)	1.03	0.87–1.21, p = 0.73	0.84–1.26, p = 1.0
	Other	161	11.8%	56 (22–170)	1.07	0.90–1.27, p = 0.42	0.87–1.32, p = 1.0
Marital status	Married/Cohabiting	311	22.8%	50 (14–214)	1.1	0.96–1.27, p = 0.19	0.93–1.31, p = 0.56
	Divorced/Separated	117	8.6%	79 (22–420)	0.94	0.76–1.16, p = 0.57	0.73–1.22, p = 1.0
	Single	804	58.9%	67 (19–276)	Reference		
	Widowed	12	0.9%	49 (12–140)	1.11	0.61–2.00, p = 0.73	0.54–2.29, p = 1.0
UK Mental Health Act	Compulsory admission within 2 weeks of first presentation	216	15.8%	21 (6–79)	2.47	2.07–2.94, p<0.001	2.07–2.94, p<0.001
Prior Diagnoses	Schizophrenia or related disorders	208	15.2%	214 (49–686)	0.4	0.34–0.48, p<0.001	0.34–0.48, p<0.001
	Psychotic depression	37	2.7%	370 (93–929)	0.73	0.52–1.04, p = 0.08	0.52–1.04, p = 0.08
	Unipolar depression without psychotic symptoms	133	9.8%	413 (128–1051)	0.4	0.33–0.48, p<0.001	0.33–0.48, p<0.001
	Anxiety disorder	78	5.7%	442 (158–901)	0.51	0.41–0.65, p<0.001	0.41–0.65, p<0.001
	Personality disorder	52	3.8%	494 (154–1025)	0.61	0.45–0.81, p = 0.001	0.45–0.81, p = 0.001
	Alcohol misuse or dependence	26	1.9%	705 (389–1341)	0.45	0.30–0.68, p<0.001	0.30–0.68, p<0.001
	Illicit drug misuse or dependence	36	2.6%	742 (320–1142)	0.43	0.31–0.61, p<0.001	0.31–0.61, p<0.001

*Model adjusted for all factors listed in this table

### Treatment delay

**Fig 2:**
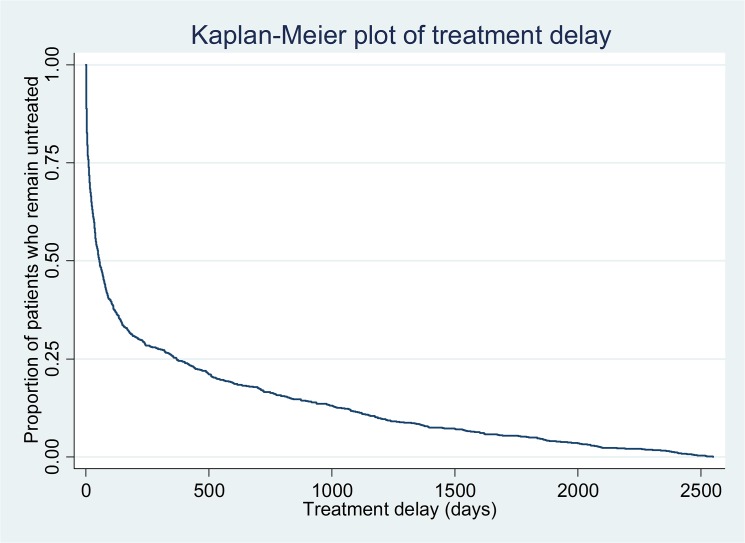
Kaplan-Meier plot of treatment delay.

Of the 1364 individuals included in this study, 1206 received appropriate treatment prior to 31^st^ December 2013 (where data were censored). 117 individuals had a treatment delay of zero days and were not included in the multivariable Cox regression analysis resulting in 1247 participants included in this analysis. The median treatment delay was 31 days (IQR 4–122). 688 individuals (57.0%) were found to have a shorter treatment delay than diagnostic delay. Kaplan-Meier analysis (Fig 2) illustrates the distribution of treatment delay over time. Table 5 shows the breakdown of treatment delay according to demographic characteristics and diagnoses recorded prior to bipolar disorder. There were no significant differences in treatment delay depending on age, gender, ethnicity or marital status. In contrast to diagnostic delay, prior diagnoses of schizophrenia (and related disorders) and psychotic depression were associated with a reduction in median treatment delay. The greatest median treatment delay was seen among people with prior alcohol misuse/dependence. Compulsory admission under the UK Mental Health Act was associated with short treatment delay. A test of proportionality of hazards (Table 6) revealed a skewed distribution of treatment delay for compulsory admission and prior diagnoses of unipolar depression without psychotic symptoms and anxiety disorder. A sensitivity analysis including only those with full covariate data (Table 7) did not result in meaningful changes to these results.

**Table 5:** Factors associated with bipolar disorder treatment delay (n = 1247)*.

Factor	Group	Number in sample	Percentage	Median treatment delay in days (IQR)	**Adjusted hazard ratio	95% confidence interval, p value	Bonferroni corrected 95% confidence interval, p value
Age (years)	16–25	314	25.2%	67 (7–532)	0.90	0.76–1.06, p = 0.21	0.73–1.11, p = 0.85
	26–35	408	32.7%	46 (9–260)	Reference		
	36–45	288	23.1%	58 (15–369)	0.96	0.81–1.13, p = 0.59	0.77–1.18, p = 1.0
	46–55	150	12.0%	59 (12–318)	1.06	0.87–1.30, p = 0.57	0.82–1.38, p = 1.0
	56–65	87	7.0%	69 (19–367)	0.81	0.62–1.05, p = 0.11	0.58–1.13, p = 0.43
Gender	Female	720	57.7%	57 (11–369)	Reference		
	Male	527	42.3%	51 (10–366)	1.09	0.96–1.23, p = 0.19	0.96–1.23, p = 0.19
Ethnicity	White	776	62.2%	73 (15–454)	Reference		
	Asian	55	4.4%	20 (3–50)	1.44	1.08–1.93, p = 0.01	1.00–2.08, p = 0.05
	Black	208	16.7%	16 (3–147)	1.14	0.96–1.36, p = 0.14	0.91–1.42, p = 0.55
	Other	163	13.1%	56 (14–241)	0.97	0.81–1.17, p = 0.78	0.77–1.23, p = 1.0
	Ethnicity not recorded	45	3.6%	83 (38-)***	0.68	0.48–0.98, p = 0.04	0.43–1.08, p = 0.16
Marital status	Married/Cohabiting	288	23.1%	38 (8–206)	1.05	0.90–1.23, p = 0.52	0.86–1.28, p = 1.0
	Divorced/Separated	113	9.1%	55 (11–369)	1.18	0.95–1.48, p = 0.13	0.89–1.57, p = 0.54
	Single	757	60.7%	67 (10–434)	Reference		
	Widowed	11	0.9%	73 (13–826)	0.42	0.21–0.84, p = 0.01	0.17–1.02, p = 0.06
	Marital status not recorded	78	6.3%	54 (14–1039)	0.89	0.67–1.17, p = 0.40	0.63–1.26, p = 1.0
UK Mental Health Act	Compulsory admission within 2 weeks of first presentation	186	14.9%	3 (1–8)	4.40	3.64–5.32, p<0.001	3.64–5.32, p<0.001
Prior Diagnoses	Schizophrenia or related disorders	183	14.7%	9 (3–75)	1.39	1.17–1.66, p<0.001	1.17–1.66, p<0.001
	Psychotic depression	35	2.8%	17 (4–109)	1.69	1.18–2.43, p = 0.004	1.18–2.43, p = 0.004
	Unipolar depression without psychotic symptoms	136	10.9%	237 (35–925)	0.75	0.61–0.92, p = 0.005	0.61–0.92, p = 0.005
	Anxiety disorder	81	6.5%	238 (24–792)	0.72	0.56–0.92, p = 0.009	0.56–0.92, p = 0.009
	Personality disorder	50	4.0%	179 (29–807)	0.92	0.67–1.26, p = 0.61	0.67–1.26, p = 0.61
	Alcohol misuse or dependence	26	2.1%	705 (231–1221)	0.59	0.38–0.90, p = 0.02	0.38–0.90, p = 0.02
	Illicit drug misuse or dependence	33	2.6%	352 (11–1104)	0.80	0.55–1.17, p = 0.24	0.55–1.17, p = 0.24

*117 cases dropped in Cox regression analysis with treatment delay of 0 days

**Model adjusted for all factors listed in this table

***75% percentile inestimable due to right censoring

**Table 6:** Test of proportionality of hazards on Schoenfeld residuals for treatment delay.

Factor	Group	Rho	χ^2^	P value
Age (years)	16–25	0.02	0.5	0.48
	26–35	Reference		
	36–45	0.07	4.9	0.03
	46–55	0.05	3.0	0.08
	56–65	0.06	4.5	0.03
Gender	Female	Reference		
	Male	0.02	0.5	0.48
Ethnicity	White	Reference		
	Asian	-0.02	0.4	0.52
	Black	-0.02	0.3	0.59
	Other	0.01	0.1	0.83
	Ethnicity not recorded	-0.01	0.0	0.84
Marital status	Married/Cohabiting	-0.03	1.11	0.30
	Divorced/Separated	0.01	0.2	0.65
	Single	Reference		
	Widowed	-0.02	0.6	0.44
	Marital status not recorded	-0.07	5.4	0.02
UK Mental Health Act	Compulsory admission within 2 weeks of first presentation	-0.13	17.4	<0.001
Prior Diagnoses	Schizophrenia or related disorders	-0.01	0.1	0.83
	Psychotic depression	-0.01	0.1	0.73
	Unipolar depression without psychotic symptoms	0.10	10.8	0.001
	Anxiety disorder	0.08	6.6	0.01
	Personality disorder	0.05	2.3	0.13
	Alcohol misuse or dependence	0.05	2.4	0.12
	Illicit drug misuse or dependence	0.02	0.5	0.50

**Table 7:** Factors associated with bipolar disorder treatment delay only including participants with complete covariate data (n = 1140)*.

Factor	Group	Number in sample	Percentage	Median treatment delay in days (IQR)	**Adjusted hazard ratio	95% confidence interval, p value	Bonferroni corrected 95% confidence interval, p value
Age (years)	16–25	287	21.0%	51 (6–403)	0.96	0.81–1.14, p = 0.66	0.77–1.20, p = 1.0
	26–35	373	27.3%	49 (9–312)	Reference		
	36–45	255	18.7%	55 (15–356)	0.99	0.83–1.18, p = 0.92	0.79–1.24, p = 1.0
	46–55	143	10.5%	59 (12–240)	1.1	0.90–1.36, p = 0.36	0.85–1.44, p = 1.0
	56–65	82	6.0%	68 (19–367)	0.8	0.61–1.05, p = 0.11	0.53–1.13, p = 0.43
Gender	Female	665	48.8%	56 (11–357)	Reference		
	Male	475	34.8%	50 (8–341)	1.09	0.96–1.24, p = 0.17	0.96–1.24, p = 0.17
Ethnicity	White	731	53.6%	73 (16–434)	Reference		
	Asian	52	3.8%	19 (3–50)	1.43	1.07–1.93, p = 0.02	1.00–2.06, p = 0.05
	Black	202	14.8%	16 (3–151)	1.13	0.94–1.35, p = 0.18	0.91–1.40, p = 0.55
	Other	155	11.4%	56 (14–260)	0.96	0.79–1.16, p = 0.65	0.76–1.21, p = 1.0
Marital status	Married/Cohabiting	281	20.6%	38 (8–212)	1.05	0.90–1.23, p = 0.54	0.87–1.28, p = 1.0
	Divorced/Separated	110	8.1%	53 (11–369)	1.21	0.97–1.52, p = 0.09	0.92–1.59, p = 0.28
	Single	738	54.1%	65 (10–403)	Reference		
	Widowed	11	0.8%	13 (73–826)	0.42	0.21–0.84, p = 0.02	0.18–0.98, p = 0.04
UK Mental Health Act	Compulsory admission within 2 weeks of first presentation	175	12.8%	4 (1–8)	4.26	3.50–5.19, p<0.001	3.50–5.19, p<0.001
Prior Diagnoses	Schizophrenia or related disorders	180	13.2%	9 (3–68)	0.42	1.19–1.70, p<0.001	1.19–1.70, p<0.001
	Psychotic depression	35	2.6%	17 (4–109)	1.71	1.19–2.45, p = 0.003	1.19–2.45, p = 0.003
	Unipolar depression without psychotic symptoms	131	9.6%	218 (35–876)	0.75	0.61–0.92, p = 0.006	0.61–0.92, p = 0.006
	Anxiety disorder	77	5.6%	238 (24–792)	0.68	0.53–0.88, p = 0.003	0.53–0.88, p = 0.003
	Personality disorder	48	3.5%	169 (26–709)	0.93	0.68–1.28, p = 0.66	0.68–1.28, p = 0.66
	Alcohol misuse or dependence	25	1.8%	832 (304–1221)	0.54	0.35–0.84, p = 0.006	0.35–0.84, p = 0.006
	Illicit drug misuse or dependence	33	2.4%	352 (11–1104)	0.78	0.54–1.14, p = 0.21	0.54–1.14, p = 0.21

*117 cases dropped in Cox regression analysis with treatment delay of 0 days

**Model adjusted for all factors listed in this table

### Variability in delays between patients

For both the delay before diagnosis, and the delay before treatment, the median and mean estimates do not convey the very large range in the length of these delays across the sample. In some patients, diagnosis and treatment were made within one day of presentation, but in others the delays extended for over five years (maximum diagnostic delay: 2110 days; maximum treatment delay: 2053 days.

## Discussion

Using a large electronic case register, we investigated the delay to diagnosis of bipolar disorder from initiation of specialist mental healthcare. The median delay to diagnosis from the point of receiving specialist mental healthcare was 62 days but varied widely (interquartile range 17–243 days). It is important to recognise that this figure does not correspond to the delay between the patient first experiencing symptoms, and diagnosis (which would be much longer), but the time between presentation to mental health services and diagnosis. Previous studies have indicated that delays to diagnosis from first experiencing symptoms of around 10 years[5–7] suggesting that there is a substantial delay from first experiencing symptoms to receiving mental healthcare. These studies obtained data from questionnaires given to patients with an established diagnosis of bipolar disorder. While they were able to elicit delays from first symptoms to bipolar disorder diagnosis, their retrospective designs are limited by the possibility of recall and information bias. It was not possible to obtain data from first symptoms to diagnosis in our study but our findings are strengthened by the use of prospectively recorded clinical data.

Our study included a maximum follow-up period of seven years (1^st^ January 2007 to 31^st^ December 2013). It is therefore possible that a longer follow-up period would have identified more people with greater diagnostic delay. However, given that the 75^th^ percentile for diagnostic delay was reached within one year follow-up, it is unlikely that a significant proportion of people had a delay to diagnosis from first presentation to mental health services extending beyond the period of this study. Although, based on previous studies, it is likely that there was a substantial additional delay from first experiencing symptoms to receiving specialist mental healthcare.

In our study, the median delay to receiving appropriate treatment for bipolar disorder was 31 days (interquartile range 4–122). The fact that the treatment delay was shorter than diagnostic delay in the majority of participants may reflect the initiation of treatment by clinicians prior to recording a formal diagnosis of bipolar disorder in electronic health records or in correspondence to other healthcare professionals. The median treatment delay in our study compares with a mean delay of 4.4 years following hospital admission in a study reported by Drancourt et al[9]. It may be that differences between our study and Drancourt et al reflect variations in the distribution of mental healthcare between primary and secondary services in different healthcare settings[29]. It is possible that in our study a substantial delay has already occurred from the time of first seeking help in primary care services to being referred to secondary mental healthcare services (resulting in a relative reduction in delay to diagnosis after presenting to secondary mental healthcare services) and that the threshold for initiating such a referral varies between different healthcare settings. Further research investigating diagnostic and treatment delay using records from primary healthcare services may help to elucidate whether this is the case.

We established that there was no significant association of age, gender, ethnicity or marital status with diagnostic or treatment delay. However, mode of presentation and previous diagnoses were associated with substantial differences in diagnostic and treatment delay. People who underwent compulsory admission to hospital under the UK Mental Health Act had a shorter delay to diagnosis and treatment. The reduced delay within this group may be explained by increased severity of illness at presentation which necessitates prompt treatment and facilitates diagnosis. However, it is possible this group may have experienced underlying symptoms of bipolar disorder for some time prior to presentation to specialist mental healthcare services.

Greater delays to diagnosis were seen among individuals with other psychiatric diagnoses recorded prior to bipolar disorder. The greatest delays were associated with prior diagnoses of alcohol or substance misuse disorders. This may reflect misattribution of symptoms of underlying bipolar disorder to an existing diagnostic framework rather than considering an alternative diagnosis. Furthermore, previous research suggests that people with symptoms of mania, hypomania or depression are at increased risk of developing alcohol and substance misuse disorders after initial onset of these symptoms[30,31]. These findings warrant increased awareness of the possibility of dual diagnosis of a substance misuse disorder and bipolar disorder among clinicians who see people at risk of these disorders[32]. While current UK treatment guidelines highlight the need for prompt recognition and treatment of both substance misuse[33] and bipolar disorder[27], there is a clear need for further research in order to better understand and treat comorbidity between the two disorders[34].

Our data also indicate a marked diagnostic delay in people who present with a prior history of unipolar depression or anxiety. It is possible that a true diagnosis of bipolar disorder is difficult to elicit in such people if they have no clear history of symptoms of mania or hypomania[35]. Delays to treatment were noted in people with other prior psychiatric diagnoses with the exception of schizophrenia and psychotic depression. This could be explained by the use of second generation antipsychotics to treat these disorders (which are also indicated in the treatment of bipolar disorder) and indicates that misattribution of bipolar disorder symptoms to another psychotic disorder may not significantly delay access to appropriate therapy even if diagnosis of bipolar disorder is delayed. Taken together, these findings indicate a need to be aware of bipolar disorder in the differential diagnosis of individuals with other psychiatric disorders, particularly among those with drug and alcohol misuse who may be using these substances secondary to experiencing affective or psychotic symptoms due to underlying bipolar disorder[18,30].

### Strengths and Limitations

The generalisability of our findings is strengthened because they were derived from routinely recorded clinical information that is representative of a population served by a large centre for mental healthcare. Furthermore, the data analysed in our study were recorded prospectively, thereby reducing the risk of information and recall bias. Although there were some missing data for covariates (ethnicity and marital status), sensitivity analyses including only participants with complete covariate data did not yield any meaningful differences in outcomes. Tests of proportionality of hazards did reveal skewed distribution of diagnostic delay and treatment delay for certain predictor variables. It is therefore not possible to make comparative inferences based on the relative magnitude of hazard ratios between predictor variables. However, it is still possible to draw the conclusions described previously based on differences in the direction of hazard between predictor variables. Furthermore, multiple linear regression revealed comparable results to Cox regression in the analysis of diagnostic delay.

Our study is also limited by virtue of analysing routinely recorded clinical data from secondary mental healthcare records. It was only possible to investigate factors associated with diagnostic and treatment delay from the point of receiving specialist mental healthcare in the centre investigated in our study. However, some individuals within the catchment area of the study centre may have initially presented to other healthcare services. It is therefore possible that the associations on subsequent delay reported in our study would be applicable to delays while receiving non-specialist healthcare. Further study examining clinical records from primary healthcare would help to elucidate this. Although 75% of people included in our study had been diagnosed within one year of follow-up, the follow-up period of 2007 to 2012 may not have been long enough to identify people with even longer delays to bipolar disorder diagnosis. While it was possible to obtain data on a range of demographic and diagnostic variables, it was not possible to obtain detailed diagnostic and symptom data using clinical rating scales such as the SCAN[36] or YMRS[37]. Previous studies have indicated that factors such as employment status[38], social support[39], deliberate self-harm[40] and the clinical experience of clinicians who first assess patients may play a role in determining delays to diagnosis and treatment. Although it was possible to analyse data on age, gender, ethnicity and marital status in our study, it was not possible to obtain data on other factors from routinely recorded electronic health records.

### Conclusion

In summary, our study indicates that certain individuals experience a significant delay in diagnosis and treatment of bipolar disorder after initiation of specialist mental healthcare, particularly those who have prior diagnoses of alcohol and substance misuse disorders. These findings highlight a need for further study on strategies to better identify underlying symptoms and offer appropriate treatment sooner in order to facilitate improved clinical outcomes, such as developing specialist early intervention services to identify and treat people with bipolar disorder.
